# Upper Crossed Syndrome in the Workplace: A Narrative Review with Clinical Recommendations for Non-Pharmacologic Management

**DOI:** 10.3390/ijerph23010120

**Published:** 2026-01-19

**Authors:** Nina Hanenson Russin, Carson Robertson, Alicia Montalvo

**Affiliations:** 1 College of Health Solutions, Arizona State University, Phoenix, AZ 85004, USA; 2 Alpha Chiropractic & Physical Therapy, Chandler, AZ 85248, USA

**Keywords:** upper crossed syndrome, UCS, workplace injuries, neck and back pain

## Abstract

**Highlights:**

**Public health relevance—How does this work relate to a public health issue?**

- Upper crossed syndrome, a musculoskeletal disorder characterized by a forward head posture, rounded shoulders, and scapular dyskinesis, is a major global contributor to neck pain, rated at 2450 individuals per 100,000, with shoulder pain incidence at 37.8 per 1000 individuals per year.
- This syndrome is particularly common among office workers who spend long periods working on computers, leading to high rates of absenteeism, reduced productivity, and disability.

**Public health significance—Why is this work of significance to public health?**

- Upper crossed syndrome is particularly prevalent among working-age adults, including young adults, whose symptoms may be exacerbated by a forward head posture during cell phone use.
- Left untreated, upper crossed syndrome can lead to more serious health problems, including cervicogenic headaches, thoracic outlet syndrome, and subacromial impingement syndrome.

**Public health implications—What are the key implications or messages for practitioners, policy makers, and/or researchers in public health?**

- Provider education regarding the symptoms of upper crossed syndrome can lead to an earlier diagnosis, when the condition is easily treatable by non-invasive methods such as stretching and corrective exercises.
- Improvements in workplace design to address poor ergonomics underlying upper crossed syndrome, combined with simple stretching exercises, could significantly reduce incidence, leading to a healthier and more productive workforce.

**Abstract:**

Problem Statement: Upper crossed syndrome (UCS), as first described by Janda, refers to a group of muscle imbalances in which tightness in the upper trapezius and levator scapulae dorsally cross with tightness in the pectoralis major and minor muscles, and weakness of deep cervical flexors cross ventrally with weakness of the middle and lower trapezius. Postural alterations from this dysfunction, including forward head, rounded shoulders, and scapular dyskinesis, contribute to upper-back and shoulder pain, particularly among office workers who spend long periods of the workday on a computer. Upper crossed syndrome is a significant contributor to both neck pain and shoulder pain among computer users, which have been rated at 55–69%, and 15–52%, respectively. Despite its prevalence, knowledge about UCS and its treatment remains spotty among primary care physicians. In addition, improvements in workstation ergonomics along with hourly work breaks may be considered as primary prevention strategies for UCS. Objectives: This narrative review examines and synthesizes evidence about the epidemiology and diagnosis of UCS, along with clinical recommendations for physiotherapeutic approaches to treatment. Ergonomic measures in the workplace, including changes in the design of computer workstations so that both the keyboard and monitor are at the proper heights to minimize the risk of long-term musculoskeletal disorders, are also critical. Methods: The first author, a Doctor of Behavioral Health, performed the initial literature search, which was reviewed by the second author, a PhD in sports injury epidemiology. The third author, a chiropractor and practice owner, provided clinical recommendations for stretching and strengthening exercises, which were also described in the literature. Discussion: While easily treatable when caught early, UCS may become resistant to noninvasive approaches over time, and more severe pathologies of the neck and shoulder, including impingement, thoracic outlet syndrome, and cervicogenic headaches may result. Because there is no specific ICD code for UCS, it is important for physicians to recognize the early signs, consider them in the context of workplace-related injuries, and understand physiotherapeutic strategies for symptom resolution.

## 1. Introduction

Upper crossed syndrome (UCS) as first described by Vladimir Janda (1928–2002) refers to a group of muscle imbalances in which tight upper trapezius and levator scapulae dorsally cross with tight pectoralis major and minor muscles and weak deep cervical flexors ventrally cross with weak middle and lower trapezius muscles [1]. A recent systematic review and meta-analysis reported the prevalence of UCS at 0.38 [2], with UCS as a major contributor to the global prevalence of both neck pain, rated at 2450 individuals per 100,000 [2], and shoulder pain, incidence at 37.8 per 1000 persons per year [3]. If left unaddressed, the rounding of shoulders in UCS can result in excessive thoracic kyphosis- an excessive outward curvature of the upper back [4]. In turn, this kyphosis can lead to scapular dyskinesis or dysfunctional anatomy and movement of the scapulae [5,6], resulting from the abnormal surface contour. Scapular dysfunction develops over time, with a frequent cause being poorly designed computer workstations. Prevalence rates of neck pain among computer users in offices has been rated at 55–69% [7], and shoulder pain within the same group has been rated at 15–52% [7]. The risk for neck and shoulder pain increases with computer use as little as 3 h daily and is significant for computer use over 42 hours per week [8]. Downstream effects include increased risk of impingement injuries, thoracic outlet syndrome, and cervicogenic headaches, all of which result in higher-than-normal absenteeism [9,10].

### Objectives

The purpose of this narrative review is to examine the relationship between UCS and poor workplace ergonomics, particularly computer workstations. Because UCS is best described as a functional musculoskeletal disorder that develops over time, it is frequently overlooked until it reaches the critical stage, at which point it may be accompanied by the other neck and shoulder conditions described above. The earlier these conditions are recognized and diagnosed, the greater the likelihood of successful treatment using non-invasive methods.

Modifications to computer workstations and administrative oversight of work breaks for stretching and corrective exercises are critical for preventing the development of UCS among office workers. These considerations may be conceptualized using the NIOSH Hierarchy of Controls framework [11]. Within this five-level framework, the third level (engineering controls) refers to materials and processes related to hazardous conditions, while the fourth level (administrative controls) refers to the way work is performed. In this case, the third level would include ergonomic modifications to ensure proper computer monitor and keyboard placement, while administrative controls would include worker education and training for the prevention of UCS symptoms.

This review concludes with clinical recommendations for diagnostic testing and treatment of UCS. Treatment plans include corrective exercises; postural evaluations and correction; stretching of shortened, tightened muscles; and strengthening of weakened muscles. It is important that patients perform the recommended exercises in the home and workplace, as well as work with employers to modify workplace ergonomics that have contributed to these muscle imbalances.

## 2. Methods

The objective of this literature review was to investigate the correlation between upper crossed syndrome and workplace-related shoulder, neck, and back pain related to computer use, as well as provide recommendations for non-pharmacologic (i.e., physiotherapeutic) treatment. This review is not intended to be a systematic search of the literature nor a complete mapping of the evidence (scoping review) in one particular area. In May 2025, the first author searched the following databases for studies on the relationship between computer use and the development of UCS; incidence and prevalence of upper crossed syndrome, as well as neck, back, and shoulder pain; and downstream musculoskeletal disorders including thoracic outlet syndrome, subacromial impingement, and cervicogenic headache: Google Scholar, PubMed, Scopus, CINAHL, and ProQuest (library subscription). Search terms included “upper crossed syndrome” OR “UCS” AND “shoulder pain” OR “upper back pain” OR “neck pain” AND “computer use”. She also searched Google Scholar for the terms “shoulder impingement” OR “thoracic outlet syndrome”, OR “cervicogenic headache” AND “upper crossed syndrome” OR “UCS”. The second author, an injury epidemiologist and certified trainer, reviewed the extracted articles, edited this manuscript, and contributed sections on thoracic kyphosis, postural evaluation, and correction. Appendix A provides the search thread utilized for PubMed.

Inclusion criteria included English-language peer-reviewed studies, with no lower date limit. The authors also utilized several books written by experts in the diagnosis and treatment of upper crossed syndrome, as well as on the Janda technique. Exclusion criteria included non-English-language literature and conference abstracts. Following the literature search, the second author (CR), a chiropractic physician and owner of a chiropractic and physical therapy practice, made clinical recommendations for recognizing, diagnosing, and treating UCS and related conditions using non-pharmacologic non-invasive methods. The clinical recommendations in this review should be considered expert commentary as opposed to systematic review evidence.

## 3. Results

Table 1 summarizes the extracted studies, populations under review, and therapeutic focus.

## 4. Findings

### 4.1. Epidemiology of UCS-Related Pain

Shoulder pain is estimated to be the third most common musculoskeletal disorder presenting in primary care, with 50% of individuals who report shoulder pain still experiencing symptoms 6 months after the initial visit [3]. A systematic review of data from 61 studies conducted between 1991 and 2020, including data from low-, middle-, and high-income nations, reported a mean community prevalence of shoulder pain at 16%, with estimates ranging from 10.8 to 55.2% and incidence estimates ranging from 7.7 to 62 cases per 1000 person-years in studies reporting over 12 months or more [3]. Estimates were consistently higher for women than men and were highest in high-income nations [3].

The age-standardized rate of point prevalence for neck pain, according to the 2017 Global Burden of Disease study, was 3551 per 100,000 individuals, with the total global prevalence at 288.7 million and 65.3 million incident cases in 2017 [29]. Although the rates of point prevalence have not changed over the measured period (1990–2017), the number of prevalent cases and incident cases has increased, which may reflect aging of the population [29]. The burden was higher among women than men, and higher in areas with higher socioeconomic indices [29].

### 4.2. UCS in the Workplace

Eight of the extracted studies reported on neck and shoulder pain in the workplace [7,9,10,13,18,20,25,35], implicating two factors: poor posture while performing office work and poor workstation design. Studies on the association between chronic neck pain and work absenteeism [9,10] reported that chronic neck pain, which may be associated with chronic migraine, is a significant cause of disability and absenteeism, with chronic neck pain and migraine reported by 12.3% of a cohort of 8232 Spanish workers [10] and 11.3% of Canadian workers [9]. Furthermore, workers reporting neck and back pain were 44% more likely to be absent for more than 30 days over a one-year period than those who did not report neck pain [10].

### 4.3. Postural Abnormalities

Among those studies examining postural abnormalities related to extended computer work among office workers, two studies specifically mention scapular dyskinesis as an underlying problem [13,25], while a third study describes excessive stress on Type 1 muscle fibers during work at computer terminals and that a greater downward tilt of the head when using a monitor can lead to pain in the neck and shoulders [35]. In a physical evaluation of 109 office workers who used computers routinely as part of their jobs, 98 (89.9%) were found to have scapular dyskinesis [25]. Those with scapular dyskinesis reported higher ratings of neck disability and neck and shoulder pain than those who did not [25].

### 4.4. Neck Pain

Office workers who report chronic neck pain frequently experience concurrent scapular dyskinesis, although that condition may or may not initially cause pain, depending on its etiology [37]. A systematic review reported the pooled prevalence estimate of upper crossed syndrome in persons experiencing neck pain at 0.35, with a higher prevalence in women than men [2]. A case–control study comparing 44 patients with neck pain due to computer use against 44 workers who did not report neck pain noted that those with pain had greater cervical spine flexion than those who were pain free and that there was a significant correlation between cervical spine flexion and scapular dyskinesis in those with neck pain [13]. A cross-sectional study of 340 individuals with neck pain in Islamabad [12] reported the occurrence of UCS at 24.1% and poor posture at 42.06%.

### 4.5. Headaches

Furthermore, cervical proprioception and scapular dyskinesis can lead to other symptoms, including cervicogenic headaches. A case study of a 56-year-old male writer having constant one-sided headaches for 5 years found upper crossed syndrome as the underlying cause of the chronic headaches [26], with physical examination revealing decreased cervical range of motion, rounded shoulders, and trigger points in the levator scapulae and upper trapezius muscles. Treatment with chiropractic adjustment, McKenzie exercises and interferential stimulation, myofascial release, and stretching resolved the headaches. A systematic review of individuals experiencing chronic primary headaches (headaches not caused by other underlying medical conditions) compared to asymptomatic controls found moderate-to-strong evidence that forward head posture as part of upper crossed syndrome was an underlying cause [16]. In addition, forward head posture has been associated with modifications in resting-state brain function in a cross-over study of 33 young computer users [21]. Participants utilized computer terminals for more than 6 h per day and had a negative history of underlying musculoskeletal, neurological, or psychiatric disorders. EEG measurements taken during neutral and forward head postures revealed significant increases in gamma oscillations in the frontal and parietal regions of the brain in patients with forward head postures, suggesting that forward head posture acts as a stressor on the brain during rest [21].

It is important to note that these musculoskeletal abnormalities may mimic cervical radiculopathy [38]. In addition, cervicogenic headaches may also result from the interaction of musculoskeletal dysfunction as described within upper crossed syndrome and cervical radiculopathy, with the diagnosis involving both MRI and EMG imaging [38]. In addition, functional testing of the scapular stabilizers, cervical flexors, and extensors may provide some answers [38].

### 4.6. Workstation Design and Work Habits

Both workstation design and work patterns can contribute to the development and maintenance of upper crossed syndrome [7,18,20]. A study of 612 primarily male young office workers found that improper monitor placement (too low) was related to chronic neck pain, while resting the arms on the desk and improper placement of the keyboard and mouse contributed to shoulder pain [20]. A cross-sectional study of slightly older office workers (mean age: 37) found a correlation between the amount of time spent daily using computers and the severity of neck, back, and shoulder pain, with individuals who used computers over 7 h per day having higher pain levels in all regions [7]. In addition to kyphotic posture, individuals who sat at the computer with an unsupported, upright posture experienced more pain than those using ergonomic desk chairs. A review of 39 epidemiological studies found that keyboard placement and the percentage of overall work time performed on a computer contributed to musculoskeletal pain. Both head rotation and keyboard height (above the elbows) were significantly associated with musculoskeletal pain in the neck, shoulders, hands, and arms.

### 4.7. Workstation Standards

Workplace standards, including recommendations for chair design, back support, leg space, computer monitors and keyboards, are included in the European International Organization for Standardization, ISO-9241 [39]. In the United States, OSHA offers an easily accessible tool for positioning computer components, as well as guidelines for the workplace environment [40]. For a complete overview of global ergonomic workplace standards, see Woo et al., 2016 [39]. The following is a brief summary of recommendations for computer and monitor placement.

### 4.8. Monitor Position

Generally, guidelines recommend that the computer monitor be placed at or below eye level to prevent eye strain or neck pain [39], with some sources recommending a position of 15–25 degrees below eye level. In addition, studies recommend placing the computer monitor closer to the keyboard to reduce the amount that users may need to shift their eyes between the two devices [41]. OSHA recommends positioning the monitor straight ahead (not further than 35 degrees to the left or right) and slightly below eye level [40]. Lighting is also a consideration, since inadequate ambient lighting may result in computer users straining to see the monitor. Distance from the monitor varies by different standards, ranging from 35 to 100 cm [39].

### 4.9. Keyboard Position

The keyboard should be at or slightly below elbow height so that the elbows are bent at a 90-degree angle when the fingers are on the keyboard [39,40,41].

### 4.10. Risk Reduction and Treatment Strategies

Strategies for the physiotherapeutic treatment of UCS are summarized in Table 2.

All of the above-described studies focus on the resolution of muscular imbalances between shortened, tight muscles, including the upper trapezius, levator scapula, suboccipitals, and pectoralis major and minor; and weakened elongated muscles, including deep cervical flexors, rhomboids, and lower trapezius, typically seen in UCS [1]. Manual-therapy approaches include instrument-assisted soft-tissue mobilization [14,22], myofascial release [14,24], the muscle energy technique [4], and proprioceptive neuromuscular facilitation [24]. Manualized approaches include open- and closed-kinetic-chain exercises [6], the Comprehensive Control Exercise Program (CCEP) [30], Kendall and McKenzie exercises [22], and Daoyin training [19]. Postural re-education and awareness training [4,15,17,19] may take place concurrently with strengthening and stretching, along with scapular stabilization [4,6,15]. Finally, the use of both elastic and rigid taping is mentioned to support key muscle groups and create awareness of correct posture [32]. In addition to traditional free weights, two studies mention the use of resistance bands [33,34], which have the advantages of being inexpensive and providing graded tension.

## 5. Discussion

### 5.1. Global Scope of UCS

Because UCS is considered a syndrome without a specific etiology, there is no ICD-10 code, with the closest approximation being R29.3 for “abnormal posture” [42]. For this reason, data regarding the global incidence and prevalence of UCS specifically are lacking. The best approximations may be derived from statistics for neck and shoulder pain. In 2012, neck pain was responsible for missed work among 25.5 million Americans, with neck and lower-back pain accounting for 134.5 billion USD in healthcare spending in the US [43]. A study of Korean claims data for shoulder pain between 2010 and 2019 revealed an upward trend in the total number of patients and costs for shoulder disorders, from 35,798 patients and 5,485,196 USD in claims in 2010, to 42,558 patients and 11,522,543 USD in 2019 [44]. During this time, opioid prescriptions for shoulder-related pain tripled.

### 5.2. Evidence for Correlation Versus Causation

Despite research suggesting a correlation between computer use and neck and shoulder pain, both primary symptoms of UCS, causation has been more difficult to prove. For example, an older systematic review of the literature found moderate evidence for a positive association between mouse use and hand–arm symptoms and weaker evidence for neck–shoulder symptoms [45]. However, the authors believed that the evidence was insufficient to support a causal relationship. A second literature review on neck pain associated with computer use led to similar conclusions [46]. Finally, a cross-sectional analytic study of neck, shoulder, hand, and back pain among newspaper office workers found that frequent computer use increased the risk of developing musculoskeletal disorder, but it did not address causation [47]. An underlying problem is heterogeneity among studies, as well as failure to tease apart physiological and psychosocial factors contributing to these symptoms [48].

However, the fact that symptoms of UCS are prevalent among office workers who use computers for extended periods suggests the importance of a functional approach, including evaluation of the work environment, static postural assessments, and dynamic assessments of upper-body function. With regard to ergonomic factors, simple adjustments to desk height, monitor height, and keyboard height (see workstation standards mentioned above) may reduce the likelihood that individuals who spend the greater part of each workday at their computer workstations will develop chronic neck or shoulder pain [7,18,20]. There is limited evidence for sit–stand desks as a strategy for reducing neck and shoulder pain, with a small randomized controlled trial in Japan (*n* = 74) finding statistically significant reductions in neck and shoulder pain following the three-month intervention [49]. A small qualitative study in Australia (*n* = 18) found mixed responses to sit–stand desks, with several workers reporting improvements in back and neck pain, while one worker reporting that the standing desk exacerbated his sciatica [50]. In all cases, individuals exhibiting symptoms of UCS should also engage in corrective exercises and stretching (see clinical recommendations below) to correct the underlying muscle imbalances.

### 5.3. Downstream Musculoskeletal Pathology

Downstream musculoskeletal disorders are also a concern, including subacromial impingement syndrome [32,34], thoracic outlet syndrome [27], rotator cuff tendonitis and/or ruptures [6], and vertebral fractures [31]. Rotator cuff injuries and subacromial impingement syndrome may originate in altered scapular kinematics [32,34], which in turn may cause the humeral head to rotate in the glenoid fossa, reducing the subacromial space [51,52]. If untreated, this can lead to bursitis, rotator cuff tendinopathy, or rotator cuff tears [53]. Scapular stabilization exercises based on a kinetic-chain approach produced significant improvements in pain and disability in a randomized controlled trial of 30 patients diagnosed with subscapular impingement and scapular dyskinesis.

Thoracic outlet syndrome (TOS) may result from tightening and scarring of the scalenes, which occur during the development of UCS [27]. There are three types of TOSneurogenic, vascular, and arterial- of which the neurogenic variety (NTOS) is the most common, accounting for over 80% of cases according to a recent epidemiological study [54,55]. Because NTOS may present with symptoms such as numbness in the hands or fingers that seem unrelated to postural abnormalities, diagnosticians may not suspect muscular imbalances as the underlying cause [27]. However, hypertrophy and/or shortening of the scalene muscles has been attributed to 70% of TOS cases, as opposed to 30% from congenital abnormalities, such as ectopic cervical ribs [55]. A correct diagnosis can therefore lead to less invasive treatments, including periscapular muscle strengthening and postural exercises [27].

## 6. Limitations

The scope of this review is limited to populations most at risk (adults who use computers extensively in the workplace), workplace risk factors, and workplace modifications. The focus was on functional (as opposed to pharmacologic) approaches to relieving symptoms and discomfort associated with UCS. The third author contributed the clinical recommendations, which were based on two decades in practice and extensive experience with working-age and older adults experiencing symptoms of neck and upper-back pain.

### 6.1. Clinical Recommendations

**Figure 1:**
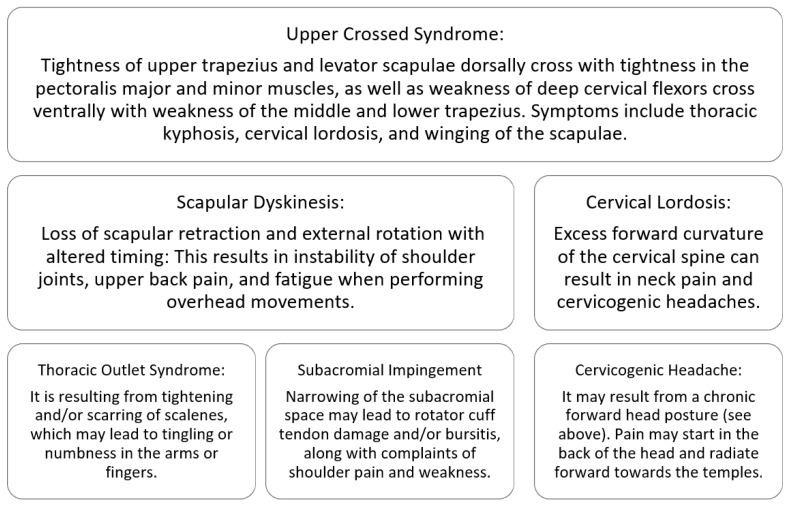
Upper crossed syndrome and related musculoskeletal disorders.

Figure 1 summarizes the presenting symptoms of upper crossed syndrome and downstream effects. Clinicians may notice the following signs: rounded and elevated shoulders, a forward head position, kyphosis, and winging of the scapulae [4,6]. Patient-reported symptoms may include chronic upper-back and/or neck pain; frequent headaches [26]; pain and weakness in the shoulders, particularly when performing overhead movements; and possibly tingling or numbness in the arms, hands, and fingers [55].

**ICD-10 Codes**

The following ICD-10 codes may be useful for reimbursement of treatment for musculoskeletal disorders described herein:

- G54.0, brachial plexus disorders (thoracic outlet syndrome)
- G44.86, cervicogenic headache
- M25.519, chronic shoulder pain (unspecified shoulder)
- M25.31, shoulder instability (unspecified shoulder)
- R53.82, chronic fatigue
- M54.2, cervicalgia.
- R29.3, abnormal posture
- M40.202, cervical kyphosis.

### 6.2. Patient’s Self-Described Symptoms

Patients may report stiffness and tightness across the neck and scapula, with discomfort worst early in the morning and late in the day. Pain at the base of the skull may progress to a tension-type headache. These headaches are more common late in the day [28].

### 6.3. Clinical Static Postural Assessment

Static postural assessment can be used to identify the presence of UCS. These assessments employ a plumb line and grid chart to allow a clinician to identify deviations from ideal postures based on standard landmark alignments. Typically, an assessment is performed in the following order: the posterior view, lateral views, and the anterior view [56]. The lateral views are the most critical for identifying the presence of UCS. An individual with UCS will demonstrate a forward head posture and rounded shoulders [4]. In the lateral view of the assessment, the individual will present with the tragus of the ear and the acromion of the shoulder anterior to the plumb line. While additional procedures can be used to quantify excessive cervical lordosis and excessive thoracic kyphosis, these deviations can also be observed from the lateral view.

### 6.4. Workplace Modifications

Recommended changes in workstation ergonomics include elevating all screens to eye level and ensuring that the elbows are bent at 90 degrees while maintaining a straight or relaxed wrist when using the keyboard [18]. If the individual uses multiple screens, it is a good idea to shift positions during the day to avoid excessive cervical flexion in either direction. Standing breaks every hour help relax the neck and shoulders. In addition, the second author (Robertson) recommends performing a short series of exercises at the desk, spaced 20–30 min apart, as opposed to scheduling a separate exercise session at the end of the day. A phone alarm can serve as a reminder to perform the exercises.

### 6.5. Neck Stretches

Neck stretches are intended to reduce tightness in the upper trapezius, suboccipital, and scalene muscles while strengthening deep cervical flexors [1]. The cervical corrective exercises are loosely based on the McKenzie method [26], which was adapted by the second author (Robertson) for use with older adult patients. The patient is instructed to sit up straight in a chair, with shoulders down and relaxed. Stretches are illustrated in Appendix A. The series of stretches is as follows:

Round the upper back and tuck the chin into the chest. With the arms straight, reach as far forward as possible, lacing the fingers together in front of the chest. Hold the stretch for at least 20 s. Relax and return to an upright seated position. Tilt the head forward and lace the fingers on the back of the head. Gently pull the head forward. Place the right hand near the back left corner of the head and pull the head gently down towards the right knee. Then use the right hand to pull the head laterally towards the shoulder. Return to the upright seated position and use the left hand to repeat this series on the other side.

### 6.6. Shoulder Exercises

**Figure A1:**
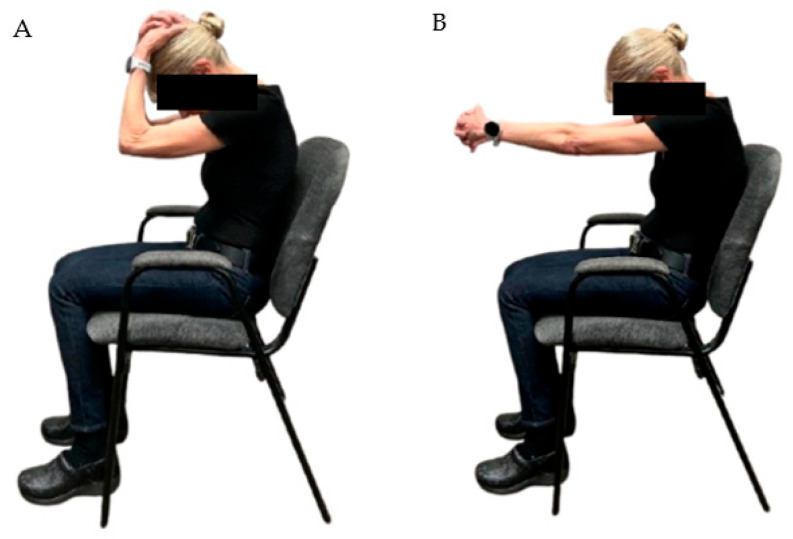
(**A**): Forward neck stretch, (**B**): Upper back stretch. Images of the first author are by Carson Robertson.

**Figure A2:**
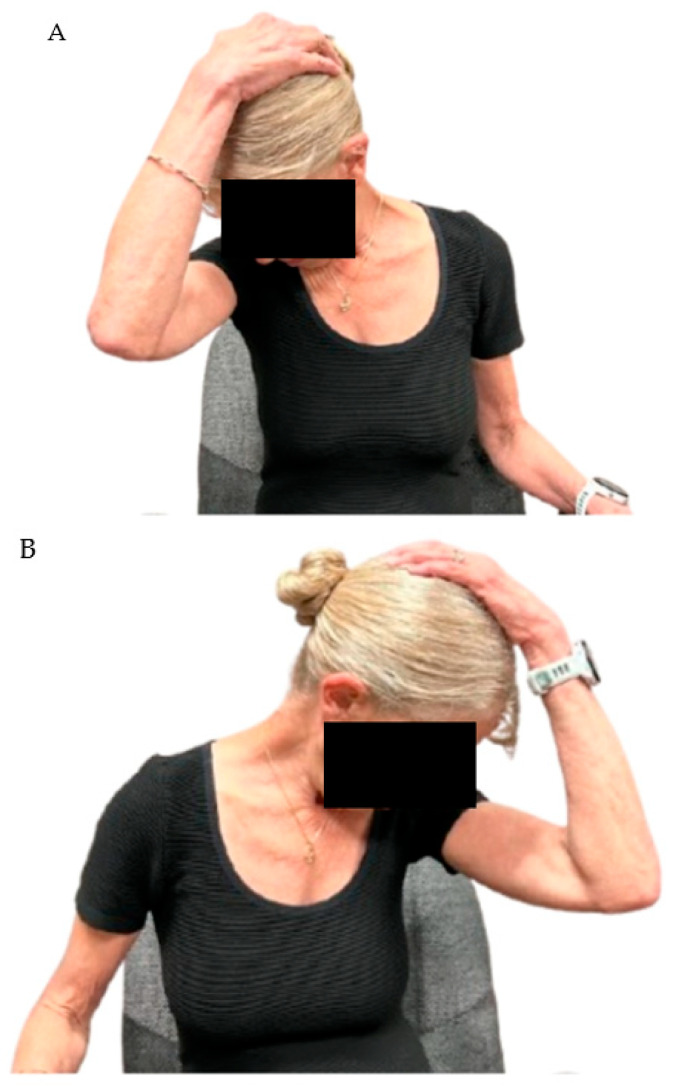
Side neck stretches. (**A**): Right side stretch, (**B**): Left side stretch.. Images of the first author are by Carson Robertson.

**Figure A3:**
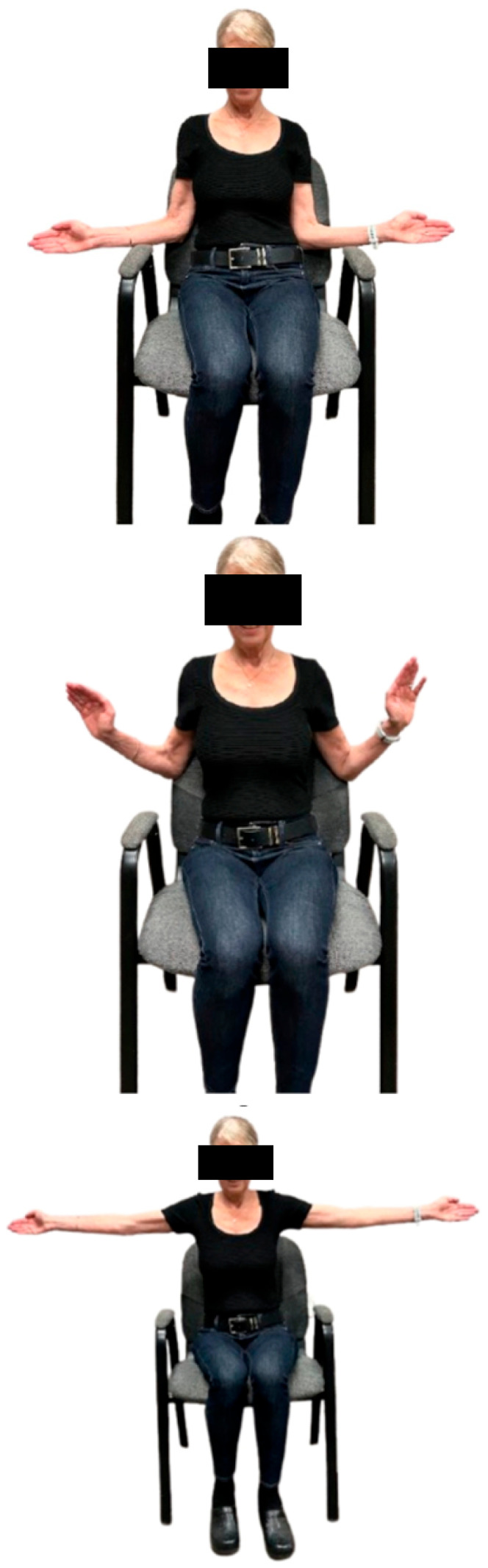

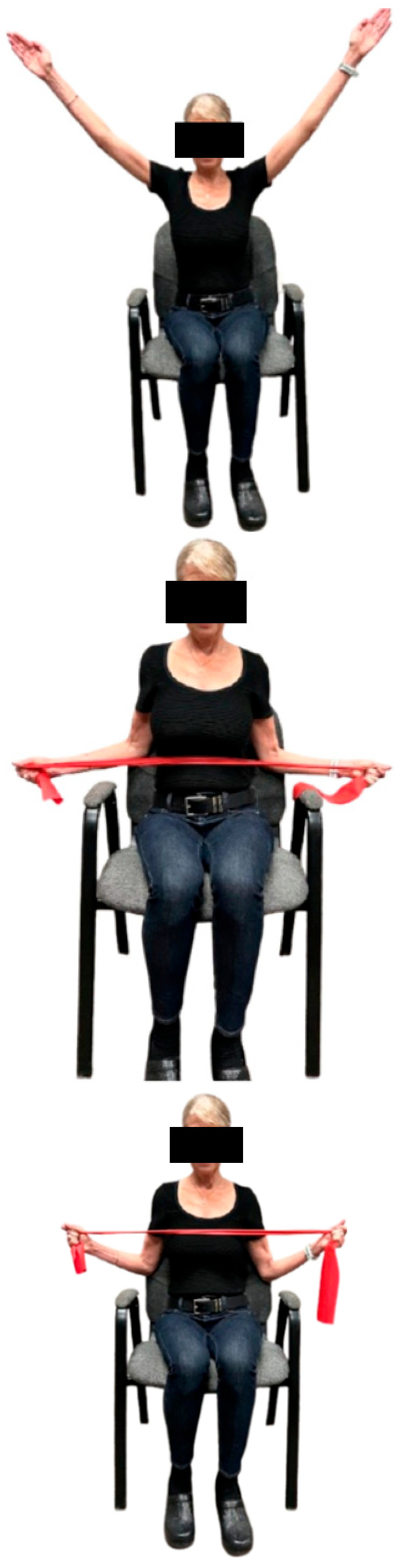

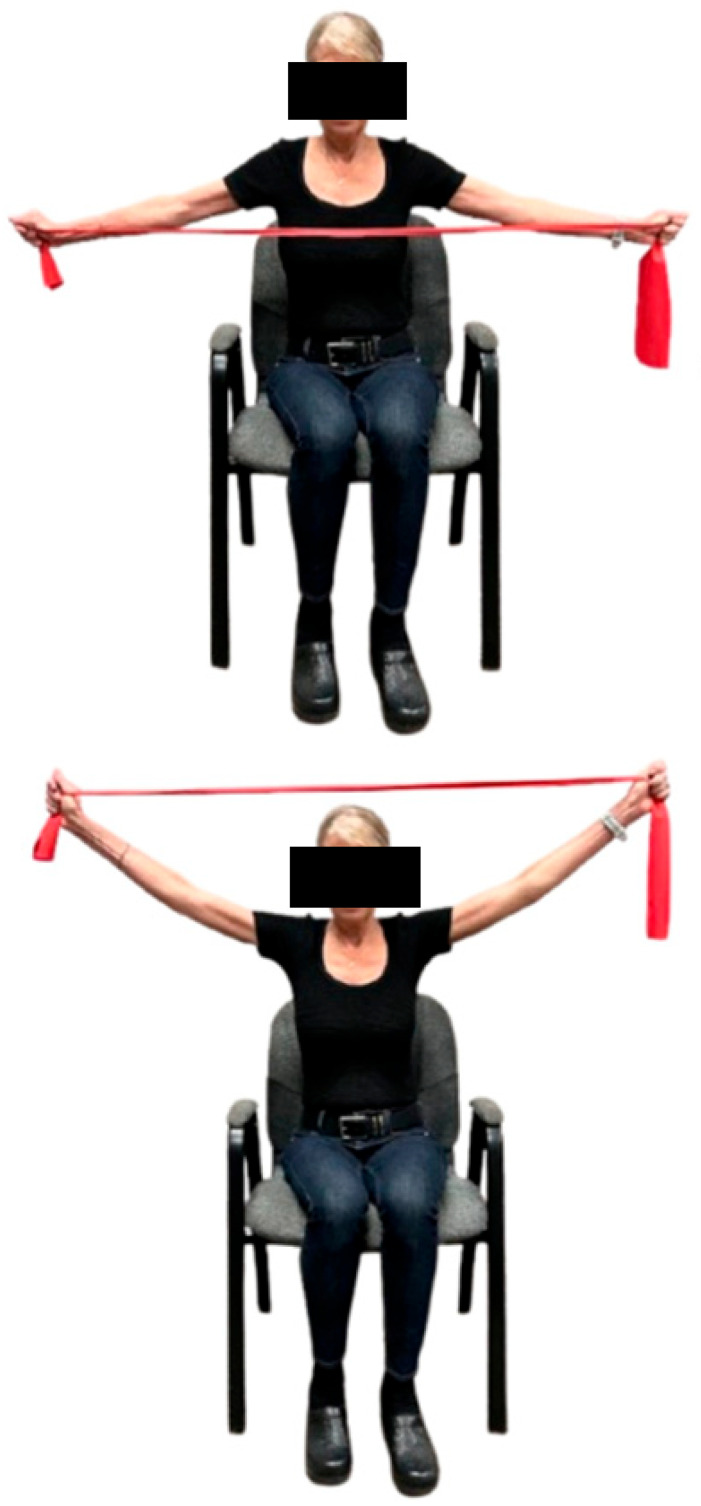
“L, W, T, and Y,” with and without TheraBand ™ resistance. Images of the first author are by Carson Robertson.

Shoulder girdle exercises strengthen scapular stabilizers including the middle and lower trapezius muscles, the serratus anterior, the rhomboids, and the levator scapulae [53]. This following series of shoulder girdle exercises is based on Brügger’s TheraBand™ exercises [1], which have been adapted by the second author (Robertson) for the workplace. The exercises can be performed while seated or standing. The shoulders should be down and relaxed. Exercises are illustrated in Appendix B (Figure A1 and Figure A2) and Appendix C (Figure A3). The series is as follows:

Squeeze the shoulder blades together, hold for 20–30 s, and relax. Return to a seated position. With the shoulders down and relaxed, elbows bent at 90 degrees, and upper arms next to the body, rotate the lower arms outward, hold to 20–30 s, and return to the starting position. Repeat this lateral rotation 2–3 times. To make this exercise more challenging, use a length of TheraBand™ about 12 inches long and holding one end of the band in each hand. TheraBands™ are available in a variety of tensions, allowing for progression as the individual becomes stronger.

Return to the seated position, with shoulders low and relaxed, upper arms against the body, and elbows bent at a 90-degree angle. Sitting up straight, move the elbows back in a rowing motion, using the back of the chair as resistance. Push the elbows into the back of the chair, hold for 10 s, and return to a relaxed position. Repeat this movement 3–4 times. Extend the arms up straight and to the sides, making a “Y” with the arms. Move the arms down and to the sides so that they are parallel with the shoulders, making a “T” with the arms. Focus on keeping the shoulders low and head erect, and squeezing the shoulder blades together while performing these exercises.

## 7. Conclusions

Upper crossed syndrome is becoming an increasingly common cause of musculoskeletal dysfunction and injury as individuals spend the greater part of their working hours at computer workstations. Successful rehabilitation involves retraining core postural muscles, taking routine standing and stretching breaks during the workday, and, to the greatest extent possible, making changes in workstation ergonomics to enable individuals to maintain proper head, neck, and shoulder alignment at their computers. Upper crossed syndrome can be particularly challenging for older adults who have a lifetime of poor postural habits to unlearn. In addition, individuals may lean forward towards the monitor to compensate for any vision problems they may be experiencing.

While physiotherapy can effectively address musculoskeletal dysfunction related to upper crossed syndrome, modifications in the workplace are equally important. This includes an evaluation of not only computer workstations but also ambient lighting to prevent unnecessary eye and neck strain, back strain, shoulder and wrist strain while working. In the US, the OSHA workstation e-tool [40] offers specific guidelines for workstation component positioning.

Future research should examine the feasibility of using the workplace stretching and strengthening routine described above for avoiding the development of upper crossed syndrome and promoting rehabilitation for individuals who are experiencing head, neck, and shoulder pain. Research studies have shown that upper crossed syndrome is a major factor underlying employee productivity, absence, and disability, making the case of integrating injury prevention into the workspace particularly important.

## Figures and Tables

**Table 1:** Extracted studies: Upper crossed syndrome and workplace computer use.

Authors, Date	Type of Study	Population	Therapeutic Focus
Asad et al., 2021 [12]	Cross-sectional survey	General population, Islamabad	Association between UCS and neck pain.
Bisen et al., 2024 [13]	Case–control	Office workers in Pune, India	Correlation between cervical proprioception, scapular dyskinesis, and neck pain.
Calik et al., 2022 [7]	Cross-sectional, mixed methods	Office workers	Risk factors for musculoskeletal disorders from office work
Chang et al., 2023 [4]	Narrative systematic review	Patients with UCS and neck pain	Overview of the treatment of UCS.
Chaudhuri et al., 2023 [14]	Systematic review and meta-analysis	Individuals with work-related UCS	Methodologic study to determine the most effective treatment strategies for UCS.
Chen et al., 2024 [15]	Systematic review and meta-analysis of RCTs	Studies in Asia, Europe, and Iran. Patients with a 3–6-month history of chronic neck pain	Effects of scapular targeted therapy on neck pain and function.
Côte et al., 2008 [9]	Review of insurance claims for workers’ compensation for neck pain	Office workers in Ontario, Canada	To measure the prevalence and incidence of neck pain in this cohort.
Elizagaray-Garcia et al., 2020 [16]	Systematic review and meta-analysis	Twelve studies of individuals with chronic primary headaches and asymptomatic controls	To compare forward head posture between individuals with chronic headaches and asymptomatic individuals.
Fernandes et al., 2025 [17]	Randomized clinical trial	52 women with chronic non-specific neck pain randomly assigned to postural re-education or specific therapeutic exercises	To determine if postural re-education is as effective as specific therapeutic exercises in reducing subjectively reported pain.
Gerr et al., 2006 [18]	Literature review	Studies investigating the relationship between computer use by time and posture and development of upper-extremity musculoskeletal disorders.	To determine if changes in ergonomics and/or hours per day of computer use could mitigate upper extremity musculoskeletal pain.
Guo et al., 2025 [19]	Randomized controlled trial	74 college students with UCS randomized to intervention and control groups	Assess efficacy of cervical and thoracic “Daoyin” training on pain, posture, function, and emotional state.
Iram et al., 2022 [20]	Cross-sectional study	Office workers who spent at least 3 h/day on computers	To determine if there is a significant relationship between computer use and complaints of arm, neck, and shoulder pain.
Jung et al., 2024 [21]	Crossover experimental protocol	33 men and women ages 18+ who were heavy computer users	To examine changes in resting-state brain function due to forward head posture.
Kaur et al., 2025 [22]	Systematic review	Systematic review of 7 systematic reviews on physiotherapeutic approaches to UCS	To investigate the relative efficacy of various physiotherapeutic strategies for UCS.
Lee et al., 2023 [23]	Experimental	50 participants ages 20–50	To use a new algorithm to classify normal and abnormal body postures during computer use.
Lucas et al., 2022 [3]	Systematic review	Data from 61 studies including low-, middle-, and high-income countries on shoulder pain	To determine the global prevalence and incidence of shoulder pain.
Mesas et al., 2014 [10]	Cross-sectional analysis	Data on 8283 Spanish workers	To examine the association between chronic neck, lower-back, and migraine pain, and absenteeism.
Moi et al., 2021 [24]	Narrative review	8 studies of UCS on patients ages 20–50 with UCS	To examine efficacy of modalities including myofascial release, exercise, stretching and strengthening, TENS, and IFT on treating UCS.
Moon & Kim, 2023 [25]	Cross-sectional, single-blind study	Office workers ages 20–50, working at least 40 h/week	To examine the relationship between scapular dyskinesis and neck and shoulder pain.
Moore, 2004 [26]	Case study	56-year-old male writer	To examine the relationship between UCS and cervicogenic headache.
Panagiotopoulos & Crowther, 2019 [6]	Summary of clinical assessment	N/A	To explain the anatomy and kinematics of the scapula, biomechanics, pathological processes, and rehabilitation.
Panther et al., 2022 [27]	Narrative review	N/A	To review diagnostic tests and treatment for thoracic outlet syndrome
Pathan et al., 2022 [28]	Narrative review	N/A	Assessment of upper crossed syndrome symptoms and a structured exercise program
Safiri et al., 2020 [29]	Systematic analysis	General population living with neck pain across 195 countries	To review data on the incidence and prevalence of neck pain from the 2017 Global Burden of Disease study.
Seidi et al., 2020 [30]	Randomized controlled trial	24 men with UCS	To investigate the efficacy of a corrective exercise program in men with UCS.
Sepehri, et al., 2024 [31]	Systematic review and meta-analysis	22 studies on the effect of therapeutic exercises on forward head posture	To examine the efficacy of specific strengthening and stretching exercises on improving the kinematics of individuals with UCS.
Takeno et al., 2019 [32]	Systematic review	7 studies	To examine therapeutic interventions for scapular kinematics and disability in patients with subacromial impingement.
Tang et al., 2024 [33]	Multi-center randomized controlled trial	90 patients with scapular dyskinesis	To compare the efficacy of scapular stabilization exercises with conventional exercises for treating shoulder pain.
Turgut et al., 2017 [34]	Randomized controlled trial	30 outpatients with scapular dyskinesis	Comparison of a scapular stabilization exercise protocol with conventional exercises for addressing kinematics, disability, and pain.
Wahlström, 2005 [35]	Narrative review	Computer users with musculoskeletal disorders	To examine the association between work environments, psychosocial factors, and physical load with musculoskeletal disorders related to computer work.
Xu, 2024 [2]	Systematic review and meta-analysis	7 studies involving 3722 participants	To examine the prevalence of UCS
Yaghoubitajani et al., 2022 [36]	Randomized controlled trial	45 subjects ages 30–45 assigned to online or workplace exercise groups and a control (no exercise intervention).	To examine the efficacy of online vs. workplace exercise interventions in reducing UCS symptoms, compared to the control group.

**Table 2:** Physiotherapeutic strategies for UCS.

Authors/Date	Treatment Recommendations
Chang et al., 2023 [4]	Posture correction, cervical or scapular stabilization, activation/strengthening of inhibited muscles (deep neck flexors), the muscle energy technique, and stretching of shortened muscles (scalenes, upper trapezius, and levator scapulae)
Chaudhuri et al., 2023 [14]	Instrument-assisted soft-tissue mobilization and myofascial release
Chen et al., 2024 [15]	Scapular correction and stability exercises, elastic-band therapy, cervical stabilization, and postural correction
Fernandes et al., 2025 [17]	Global postural education and cervical-specific therapeutic exercises for addressing neck pain in persons with UCS
Guo et al., 2025 [19]	Postural assessment followed by “Daoyin” training that mimics the movements and postures of natural animals including turtles, swans, rocs (mythical birds of prey), and tigers to correct for forward head posture and rounded shoulders
Kaur et al., 2025 [22]	Kendall exercises, comprehensive corrective exercises, stretching, strengthening, cervical segmental mobilization, McKenzie traction, and instrument-assisted soft tissue mobilization
Moi et al., 2021 [24]	Stretching, strengthening, myofascial release, neuromuscular re-education, and proprioceptive neuromuscular facilitation (PNF)
Panagiotopoulos & Crowther, 2019 [6]	Scapular orientation exercise, education regarding neutral spine position, re-engagement of paraspinal-stabilizing muscles, and open-chain and closed-kinetic-chain exercises
Pathan et al., 2022 [28]	Postural evaluation, muscle length testing, muscle strength evaluation, and corrective exercise
Seidi et al., 2020 [30]	Comprehensive Control Exercise Program (CCEP), a program with a multifaceted focus on muscle activation, movement patterns, and postural alignment
Sepehri et al., 2024 [31]	To address forward head posture, exercises focused on stretching shortened neck muscles (SCM, levator scapulae, scalenes, and pectoralis major), and strengthening deep neck flexor muscles (longus colli, longus capitus, and anterior scalenes).
Takeno et al., 2019 [32]	Elastic and rigid taping, thoracic spine manipulation, scapular mobilization, and strengthening exercises
Tang et al., 2024 [33]	Pectoralis minor stretching; serratus anterior, middle and lower trapezius strengthening; teres major strengthening; rhomboid strengthening; upper trapezius and levator scapulae stretching. Scapular stabilization exercises utilizing elastic bands and dumbbells for resistance.
Turgut et al., 2017 [34]	Shoulder girdle stretching and strengthening based on a kinetic-chain approach. Scapular stabilization exercises included wall slides with squats, wall push-ups, resisted scapular retraction, lawnmower with diagonal squats, and resistance exercises with graded elastic bands. Stretching included the pectoralis minor, levator scapulae, and latissimus dorsi.
Yaghoubitajani et al., 2022 [36]	Workplace exercises focus on scapular retraction, arms in the T and W shapes, and overhead arm exercises. Online exercises are standing and seated, scapular retraction and depression, external rotation, and arms in the T and W positions with TheraBand™ for added resistance.

## Data Availability

No new data were created or analyzed in this study. Data sharing is not applicable to this article.

## Appendix A. Search Strategy

The following search string was utilized for PubMed: (((((upper crossed syndrome) OR (UCS)) AND (upper back pain)) OR (shoulder pain)) OR(neck pain)) AND (computer use).
